# Beyond traditional metrics: the holistic role of multisource feedback in comprehensive dental clinical education

**DOI:** 10.3389/fmed.2026.1845281

**Published:** 2026-05-28

**Authors:** Yanting Liao, Xunmin Xu, Yong Jiang, Cong Fan, Yi Zhang, Na An

**Affiliations:** 1 Department of General Dentistry II, Peking University School and Hospital of Stomatology & National Center for Stomatology & National Clinical Research Center for Oral Diseases & National Engineering Research Center of Oral Biomaterials and Digital Medical Devices & Beijing Key Laboratory of Digital Stomatology & NHC Key Laboratory of Digital Stomatology & NMPA Key Laboratory for Dental Materials, Beijing, China; 2 Department of Oral Emergency, Peking University School and Hospital of Stomatology & National Center for Stomatology & National Clinical Research Center for Oral Diseases & National Engineering Research Center of Oral Biomaterials and Digital Medical Devices & Beijing Key Laboratory of Digital Stomatology & NHC Key Laboratory of Digital Stomatology & NMPA Key Laboratory for Dental Materials, Beijing, China

**Keywords:** 360-degree evaluation, clinical training, dental education, multisource feedback, undergraduate internship

## Abstract

**Introduction:**

With dental education progressively moving toward competency-based training models, the importance of multidimensional evaluation has continued to grow. This study introduces multisource feedback into clinical internships of undergraduate dental students for the first time.

**Methods:**

In this study, we developed a tailored 360-degree evaluation system for undergraduate clinical training. Participants included 77 students, two clinical instructors, and one nursing staff from PKUSS. A total of 913 questionnaires were distributed electronically to all eligible participants. Assessment scores were analyzed using descriptive statistics. Competency differences between the two clinical instructors were compared using a paired-samples *t*-test, while variations among multisource ratings of student competencies were examined using the Friedman test. When the Friedman test indicated a significant difference, *post-hoc* pairwise comparisons were performed using the Wilcoxon signed-rank test, with a Bonferroni-adjusted significance level set at 0.0167.

**Results:**

The overall response rate was 98.8%. The dental students achieved an average total score of 86.78, demonstrating good performance across all competency domains—professionalism, clinical competence, communication and collaboration, and learning capacity. The two clinical instructors scored 90.63 and 91.37, respectively, with no statistically significant difference between them. However, significant differences were observed among the three rater groups—clinical instructors, the nursing staff, and peers—in their assessments of students' collaboration (*p* = 0.012) and communication (p = 0.043) skills. *Post-hoc* pairwise comparisons indicated that clinical instructors rated students' collaboration skills significantly higher than both the nursing staff (9.08 ± 1.11 vs. 7.82 ± 1.18; *p* = 0.014) and peers (9.08 ± 1.11 vs. 8.18 ± 1.02; *p* = 0.016). Additionally, the nursing staff provided significantly lower ratings for communication skills compared to peers (7.98 ± 0.89 vs. 9.01 ± 1.12; *p* = 0.010).

**Conclusion:**

Multisource feedback enables a comprehensive assessment of both medical students' competencies and the teaching abilities of instructors. The students demonstrated strong professionalism; however, there is still room for improvement in areas such as comprehensive diagnosis and treatment, collaboration, and communication skills. Further exploration and improvement are needed to effectively provide targeted training, thereby improving the effectiveness of clinical internships.

## 1 Introduction

The 360-degree evaluation, also called multisource feedback (MSF), is a method that involves collecting information about an individual's competencies (such as teamwork, communication skills, decision-making, and problem-solving abilities) through surveys and questionnaires completed by multiple parties closely associated with the individual, including superiors, subordinates, colleagues, and peers (1). This approach was originally implemented in the field of human resource management and development in large corporations (2). Since its introduction into residency training programs in the 1990s, the 360-degree evaluation has become an important tool for assessing the competencies of resident physicians. It comprehensively evaluates residents' performance from various perspectives, including supervising physicians, peers, interns, nurses, and patients—all of whom work closely with the residents. The value of this method in competency assessment has been continuously validated (3–6). As a comprehensive, multi-perspective evaluation approach, it incorporates diverse dimensions of assessment, facilitating the analysis of existing issues from multiple perspectives. The results are then fed back to the evaluated individual through a structured process. It has been shown to be a feasible, reliable, and valid method for evaluating medical practice, particularly in assessing non-technical competencies such as communication skills, interpersonal abilities, collegiality, humanism, and professionalism (7–10).

The evaluation of clinical internship effectiveness for undergraduate dental students primarily focuses on assessing their clinical competencies. In recent years, assessment methods have evolved from pure clinical skill examinations to a diversified and multi-tiered framework. The current evaluation system includes both formative and summative assessments. However, it still predominantly relies on clinical patient interactions and end-of-rotation examinations, with attending physicians serving as the sole evaluators. However, with the ongoing shift toward *competency-based* undergraduate dental education, there is a growing emphasis on cultivating dental professionals with strong vocational qualities and job readiness. This approach necessitates not only mastery of theoretical knowledge but also increasing attention to the comprehensive assessment of clinical skills, practical abilities, professional ethics, and even humanistic care (11–13).

Globally, the predominant training model for clinical internships in dental education emphasizes comprehensive diagnosis and treatment (14–16), which better aligns with the evolving demands of the profession. Department of General Dentistry II at Peking University School and Hospital of Stomatology (PKUSS) was established in 2008. As a teaching base, it has undertaken the task of clinical internships for dental students for 16 consecutive cohorts. Since 2022, we have introduced a 9-week comprehensive curriculum following the discipline-specific rotations, along with a newly established *Dental Comprehensive Specialty*. This new teaching model has been consistently well-received by four consecutive cohorts of students and has shown initial success in helping students develop comprehensive diagnostic thinking and train multi-specialty clinical skills (17). However, due to the relatively short period of implementation, a thorough and systematic evaluation system is still lacking to fully assess students' integrated competencies in learning, clinical practice, communication, and other essential areas.

This study represents the first application of a 360-degree evaluation system within this new comprehensive clinical internship program for undergraduate dental students. The assessment captures multisource feedback from clinical instructors, nursing staff, and peers to evaluate trainees' competencies—including professionalism, clinical competence, communication skills, collaboration skills, and learning and improvement capability. Concurrently, student evaluations provide feedback on the teaching proficiency of clinical instructors—covering their dedication to teaching, instructional skills, and clinical expertise.

## 2 Materials and methods

### 2.1 Study participants

The study enrolled undergraduate students, clinical instructors, and the nursing staff participating in clinical internship in Department of General Dentistry II under PKUSS between August 2023 and June 2025. The student cohort consisted of 77 fifth-year and eighth-year undergraduates from the class of 2020, all of whom completed the full clinical internship in the department. The student participants were divided into nine groups, with 8–9 students per group. The two clinical instructors were attending physicians specializing in General Dentistry. One was a senior attending physician, and the other held an associate senior professional title. Both had over 10 years of clinical teaching experience.

### 2.2 Development of a 360-degree evaluation system

The teaching team of the Department of General Dentistry II developed a 360-degree evaluation system tailored for undergraduate clinical internship by reviewing relevant literature, conducting field visits, and studying the principles and methods of 360-degree evaluation. The team also drew upon existing evaluation practices from other dental specialties and referenced the 360-degree framework used in standardized residency training. Adapted to the specific requirements of comprehensive dentistry training, the resulting system is structured into two main components: evaluation on dental students and evaluation on clinical instructors (Tables 1, 2). The final version of the evaluation employed a Likert scale to quantify respondents' perceptions, allowing for nuanced measurement of attitudes and experiences while maintaining response consistency.

**Table 1:** Evaluation on dental students.

Competences (points)	Items	Points	Scoring		
			Instructors	Nurse	Peers
Professionalism (20)	Compliance, attitude, and civility	5	√	√	√
	Diligence, rigor, and initiative	5	√	√	√
	Timely submission of the internship logbook	5	√	-	-
	Attendance policy (Point Deductions): Unexcused Absence: −2 points; Tardy/Early Leave: −1 point	5	√	√	√
Clinical competence (40)	Comprehensive workup and treatment planning	15	√	-	-
	Clinical procedures	15	√	√	√
	Comprehensive medical records	10	√	-	-
Communication and collaboration (20)	Collaboration skills, particularly in patient care coordination and group-based case compilation	10	√	√	√
	Communication skills with patients, instructors, and peers	10	√	√	√
Learning capacity (20)	Self-directed learning ability	8	√	√	√
	Quality of the group-based comprehensive case report	10	√	-	-
	Timeliness of case submission	2	√	-	-

“√” indicates items that require scoring; “-” indicates items that are not scored.

**Table 2:** Evaluation on clinical instructors.

Competences (points)	Items	Points
Dedication to teaching (30)	Strong dedication and passion for teaching	10
	Demonstrates responsibility and patience	10
	Dedicated solely to teaching	10
Instructional competence (40)	Adheres to standard teaching protocols and employs a structured approach	10
	Attentive to students' progress and offers timely intervention at every crucial stage	10
	Maintains professional decorum in verbal and behavioral communication during instruction	10
	Conducts timely and thorough summarization and guidance	10
Clinical expertise (30)	Exceptional clinical competence	15
	Strong interpersonal skills in patient care	15

#### 2.2.1 Evaluation on dental students

Feedback was collected from three sources: clinical instructors, nursing staffs, and peers. The assessment criteria for students were organized into four domains: professionalism, clinical competence, communication and collaboration skills, and learning and improvement capacity. Each domain included several specific evaluation items (Table 1). Since nursing staffs and peers are not involved in certain activities—such as submission of logbooks, comprehensive examination and treatment planning, medical record documentation, or assessment of comprehensive cases—these items are excluded from their evaluations.

#### 2.2.2 Evaluation on clinical instructors

This component primarily consists of feedback provided by dental students regarding their clinical instructors. The evaluation criteria for instructors are organized into three key domains: dedication to teaching, instructional competence, and clinical expertise. Each domain comprises several specific assessment indicators (Table 2).

### 2.3 Application of the 360-degree evaluation system

This study was approved by the Institutional Review Board of Peking University School and Hospital of Stomatology (PKUSSIRB), according to the Helsinki Declaration (Approval number: PKUSSIRB-2024105199). A waiver of written informed consent was granted, and verbal consent was obtained from all participants after they were fully informed about the study purpose and procedures. Participation was entirely voluntary and completely anonymous.

This study employed a complete enumeration sampling method. A total of two instructors, one nurse staff and 77 undergraduate dental students from the Department of General Dentistry II at PKUSS were all invited. All participants were informed and consented to participate in the study. The questionnaire was administered to the entire cohort of participants during the final week of the internship program through a secure electronic survey platform, with distribution facilitated via the widely-used WeChat messaging application to ensure timely and efficient data collection. Instructions were provided uniformly, and the respondents anonymously filled out the survey.

Among 77 dental students, 38 were from the 5-year program and 39 from the 8-year program, including 31 males and 46 females. The students were randomly divided into 9 groups, with 8–9 members per group. At the end of the internship, each student was evaluated by two clinical instructors, one nursing staff, and 7–8 peers in their group. Additionally, the students in each group also evaluated both of their clinical instructors.

The development of questionnaire followed a rigorous, multi-stage process to ensure its validity and reliability. Initially, the questionnaire was conceptualized and drafted through structured focus group discussions involving domain experts in dental education, including 4 dental education researchers, 2 senior dental clinical teachers, and 2 dental education administrators, covering the disciplines of dental education, clinical dentistry, and educational management. Subsequently, it underwent comprehensive content validation by an independent panel of eight experts in the field, who evaluated the relevance (whether each item is closely related to the research objectives and dental education practice), clarity (whether the wording of each item is concise, unambiguous, and easy to understand for respondents, and appropriateness (whether the item setting is reasonable, in line with the characteristics of the research objects and the scope of the questionnaire) of each item. The validation resulted in Cronbach's α values of 0.91 and 0.88 for the scales in Tables 1, 2, respectively, which indicate good internal consistency reliability and support the content validity of the questionnaire.

### 2.4 Statistical analysis

All statistical analyses were conducted using SPSS version 26 software (SPSS, IBM Corporation, Chicago, IL, USA). Descriptive statistics were employed to analyze the scores of dental students and instructors. Quantitative data are presented as mean ± standard deviation (X ± s). A Paired-Samples *t*-test was used to compare the performance ratings between the two clinical instructors. Differences among the three rater groups (clinical instructors, nursing staff, and peers) in their evaluations on dental students' competencies were analyzed using the Friedman Test instead of ANOVA because of the three related samples rather than independent samples. All statistical tests were two-sided, with a *p*-value < 0.05 considered statistically significant. If the Friedman Test indicated a significant difference, *post-hoc* pairwise comparisons were performed using the Wilcoxon Signed-Rank Test, with the significance level adjusted to 0.0167 (approximately 0.05/3) via the Bonferroni correction.

## 3 Results

A total of 77 students (including 31 males and 46 females; 38 from 5-year program and 39 from 8-year program), 2 clinical instructors and 1 nursing staff participated in the evaluation. A total of 913 questionnaire-based rating forms were distributed and 902 were returned, yielding an overall response rate of 98.8%. Among these, 154 evaluations on dental students by clinical instructors, 77 evaluations on students by the nursing staff, and 154 evaluations on instructors by students were collected, all achieving a 100% response rate. Additionally, 517 peer evaluations among students were received, corresponding to a response rate of 97.9% (*n* = 517/528).

### 3.1 Overall results of evaluations on dental students

The dental students achieved a mean score of 18.80 in *Professionalism* (out of 20), 33.76 in *Professional Competence* (out of 40), 17.15 in *Communication and Collaboration* (out of 20), and 17.07 in *Learning Capacity* (out of 20). The total average score was 86.78 out of 100, indicating excellent performance across all competency domains. Detailed scores for each item are presented in Table 3.

**Table 3:** Results of evaluation on dental students.

Competences (points)	Items	Points	Scoring
Professionalism (20)	Compliance, attitude, and civility	5	4.91 ± 0.20
	Diligence, rigor, and initiative.	5	4.52 ± 0.51
	Timely submission of the internship logbook	5	4.59 ± 0.29
	Attendance policy (Point Deductions): Unexcused Absence: −2 points; Tardy/Early Leave: −1 point	5	4.78 ± 0.31
Clinical competence (40)	Comprehensive workup and treatment planning	15	11.90 ± 2.14
	Clinical procedures	15	12.92 ± 2.82
	Comprehensive medical records	10	8.94 ± 2.13
Communication and collaboration (20)	Collaboration skills, particularly in patient care coordination and group-based case compilation	10	8.33 ± 1.03
	Communication skills with patients, instructors, and peers	10	8.82 ± 1.31
Learning capacity (20)	Self-directed learning ability	8	7.04 ± 1.39
	Quality of the group-based comprehensive case report	10	8.23 ± 1.58
	Timeliness of case submission	2	1.80 ± 0.60

**Figure 1:**
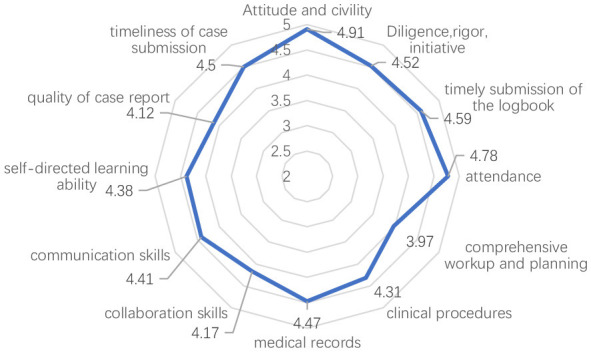
Radar chart of evaluations on dental students.

To provide a more intuitive comparison of the scores across different competencies, all ratings were converted to a five-point scale and visualized using a radar chart (Figure 1). The scores for the dental students' competencies ranged from 3.97 to 4.91 out of 5. All four items under *Professionalism* scored above 4.5. Among all the competences, “*Comprehensive workup and Treatment planning*” received the lowest score of 3.97, followed by “*Quality of the group-based comprehensive case report*” at 4.12 and “*Collaboration skills*” at 4.17.

### 3.2 Results of evaluations on clinical instructors

Clinical Instructors A and B received mean scores of 27.47 and 27.42, respectively, in *Teaching Dedication* (out of 30); 36.30 and 36.79 in *Instructional Competence* (out of 40); and 26.86 and 27.16 in *Clinical Expertise* (out of 30). Their overall mean total scores were 90.63 and 91.37 out of 100, respectively. Detailed scores for each evaluation item are presented in Table 4. No significant differences were observed between the two instructors across any of the assessed domains.

**Table 4:** Results of evaluations on clinical instructors A and B.

Competences (points)	Items	Instructor A	Instructor B	*p*
Dedication to teaching (30)	Strong dedication and passion for teaching (10)	9.24 ± 2.25	9.23 ± 2.11	0.884
	Demonstrates responsibility and patience (10)	8.71 ± 1.70	8.65 ± 1.62	0.735
	Dedicated solely to teaching (10)	9.52 ± 1.58	9.54 ± 1.55	0.698
Instructional competence (40)	Adheres to standard teaching protocols and employs a structured approach (10)	9.24 ± 1.78	9.30 ± 1.81	0.435
	Attentive to students' progress and offers timely intervention at every crucial stage (10)	9.14 ± 2.32	9.10 ± 2.17	0.782
	Maintains professional decorum in verbal and behavioral communication during instruction (10)	9.45 ± 1.98	9.47 ± 2.12	0.628
	Conducts timely and thorough summarization and guidance (10)	8.47 ± 2.21	8.92 ± 2.32	0.261
Clinical expertise (30)	Exceptional clinical competence (15)	13.38 ± 2.88	13.59 ± 2.49	0.498
	Strong interpersonal skills in patient care (15)	13.48 ± 2.35	13.57 ± 2.18	0.522

### 3.3 Comparison of evaluations on students from multisource

As shown in Table 5, a comparison of the evaluations on undergraduate dental students from clinical instructors, nursing staffs, and peers (hereinafter referred to as multisource) showed no significant differences among their ratings of the students' competences in the three items under *Professionalism*, as well as in the item *clinical procedures* (under *Clinical Competence*) and the items s*elf-directed learning capacity* (under *Learning Capacity*). However, significant differences were identified for *collaboration skills* (*p* = 0.012) and *communication skills* (*p* = 0.043).

**Table 5:** Comparison of evaluations on students by clinical instructors, nurse, and peers.

Competences (points)	Items	Instructors	Nurse	Peers	*p*
Professionalism (20)	Compliance, attitude, and civility (10)	4.86 ± 0.12	4.9 ± 0.21	4.91 ± 0.18	0.683
	Diligence, rigor, and initiative (10)	4.47 ± 0.34	4.51 ± 0.4	4.51 ± 0.61	0.527
	Timely submission of the internship logbook (10)	4.59 ± 0.29	-	-	/
	Attendance Policy (10)	4.75 ± 0.32	4.77 ± 0.41	4.82 ± 0.27	0.433
Clinical competence (40)	Comprehensive workup and Treatment Planning (15)	11.90 ± 2.14	-	-	
	Clinical Procedures (15)	13.21 ± 2.01	12.68 ± 1.91	12.87 ± 2.41	0.282
	Comprehensive Medical Records (10)	8.94 ± 2.13	-	-	/
Communication and collaboration (20)	Collaboration skills, particularly in patient care coordination and group-based case compilation (10)	9.08 ± 1.11^**a, b**^	7.82 ± 1.18	8.18 ± 1.02	**0.012** ^*^
	Communication skills with patients, instructors, and peers (10)	8.68 ± 1.23	7.98 ± 0.89^**c**^	9.01 ± 1.12	**0.043** ^*^
Learning capacity (20)	Self-directed learning ability (8)	6.82 ± 1.32	6.54 ± 1.47	7.01 ± 1.68	0.126
	Quality of the group-based comprehensive case report (10)	8.23 ± 1.58	-	-	/
	Timeliness of case submission (2)	1.80 ± 0.60	-	-	/

“-” indicates not scored.

^*^p < 0.05 among three groups. ^a^instructors vs. nurse, *p* = 0.014; ^b^instructors vs. peers, *p* = 0.016; ^c^nurse vs. peers, *p* = 0.010. Bold values indicate statistical significance with *p* < 0.05.

Using the Bonferroni correction, the adjusted significance level was set at 0.0167 (approximately 0.05/3). *Post-hoc* pairwise comparisons revealed that clinical instructors rated students' *collaboration skills* significantly higher than both the nursing staff (9.08 ± 1.11 vs. 7.82 ± 1.18, *p* = 0.014) and peers (9.08 ± 1.11 vs. 8.18 ± 1.02, *p* = 0.016). In addition, the nursing staff rated students' *communication skills* significantly lower than peers did (7.98 ± 0.89 vs. 9.01 ± 1.12, *p* = 0.010).

**Figure 2:**
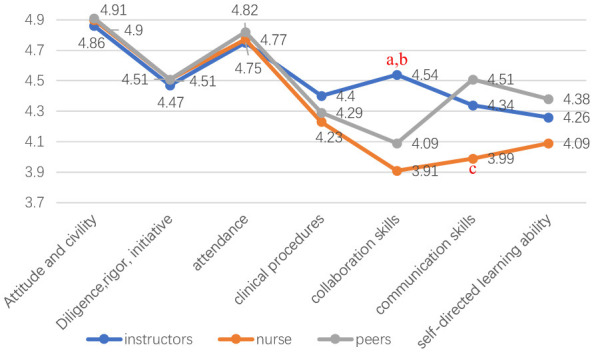
Comparison of five-point scale scores from multisource across different competencies. **(a)** Instructors vs. nurse, *p* = 0.014; **(b)** Instructors vs. peers, *p* = 0.016; **(c)** nurse vs. peers, *p* = 0.010.

To allow for a more intuitive comparison of rating differences from multisource, scores for all items were converted to a five-point scale (Figure 2). Ratings for the dental students' *attitude and civility, rigor and initiative, attendance, clinical procedures*, and *self-directed learning ability* showed no significant differences across the three groups. However, in terms of *collaboration skills*, clinical instructors provided significantly higher ratings. Conversely, the nursing staff gave notably lower scores in the domain of *communication skills*.

**Figure 3:**
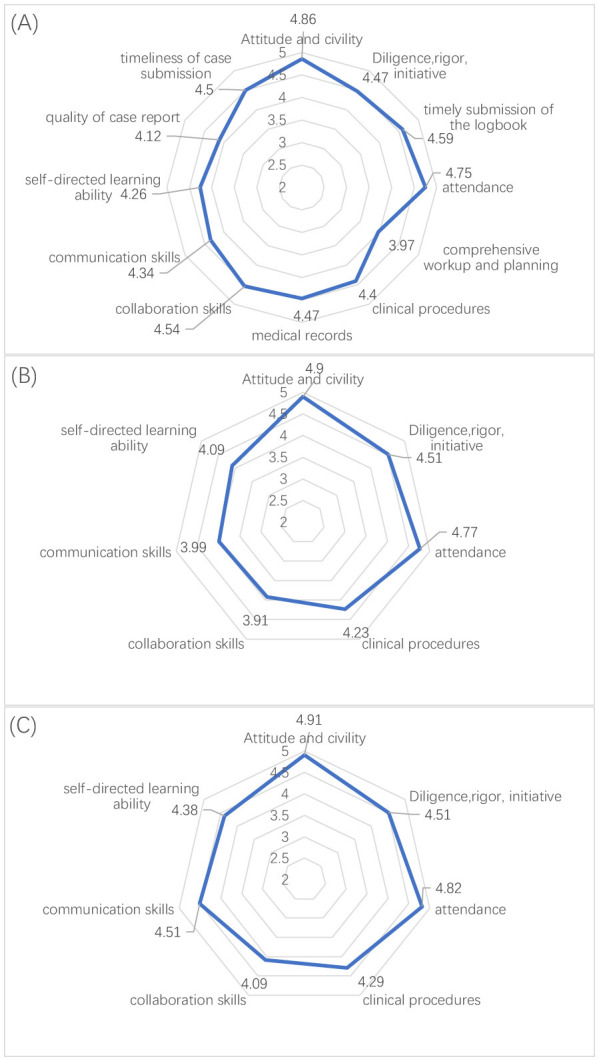
Radar chart of evaluations on dental students by clinical instructors, nursing staff, and peers. **(A)** Evaluation by clinical instructors; **(B)** Evaluation by nursing staff; **(C)** Evaluation by peers.

Based on the five-point scale scores of all evaluation items, radar charts were used to visualize the assessment results provided by clinical instructors, nursing staff and peers regarding the medical students' performance. The results indicate generally high recognition from three parties for most competencies. However, ratings below 4 were observed in a few areas: instructors' ratings for “*comprehensive workup and treatment planning*” (3.97), and nursing instructors' ratings for “*collaboration skills*” (3.91) and “*communication skills*” (3.99). All three groups consistently agreed that the dental students demonstrated excellent professionalism (all item scores > 4.5), strong clinical procedural skills, competent medical documentation abilities, and solid learning capacity. Some relative weakness was noted in the domain of communication and collaboration (Figure 3).

## 4 Discussion

The internship evaluation of dental students has shifted toward a multidimensional framework, placing equal emphasis on theoretical knowledge and clinical proficiency, as well as professional attitude, learning potential, research literacy and other core competencies required for future dental practitioners (18). In our clinical internship program, instructors intentionally foster students' comprehensive thinking and develop their ability to collect, analyze, and present comprehensive case reports. Coordination activities among students—such as assisting each other during treatments and jointly developing comprehensive case report—further enhance their collaboration and communication skills. The aim of this study was to explore the effects of clinical internship programs on the core competencies of dental students and the effectiveness of multidimensional internship evaluation.

The main results of this study indicated that: (1) instructors' intentional cultivation in the clinical internship effectively improved students' holistic thinking and their clinical ability to collect, analyze, and present comprehensive case reports; (2) peer coordination activities, including mutual assistance during treatments and joint development of case reports, enhanced students' collaboration and communication skills; (3) the adoption of multidimensional evaluation in dental student internships, which integrates assessment of professional attitude, knowledge, technical skills and learning potential, is more conducive to evaluating students' overall core competencies required for future dental practice. On the basis of these findings, the following discussion will specifically interpret the implications of each result, and elaborate on the implications for dental education and clinical internship management.

In our training model, the teacher-student ratio is maintained at 1:4–1:5, with one teacher supervising 4–5 students. Consequently, instructors cannot monitor every student continuously. Students are grouped into teams of 8–9 members for clinical training. Within each group, 3–4 students take on assisting roles during treatments, allowing them to learn through observation. At the end of the internship, each group is required to submit a comprehensive treatment planning case. This training structure requires students to exercise strong collaboration and communication skills with their peers, particularly during assisting treatment activities and developing a comprehensive treatment planning case. Furthermore, by acting as observers, students gain a closer perspective on their peers' performance in areas such as clinical procedures, patient communication, and intra-group collaboration, enabling them to provide more grounded evaluations.

Therefore, in the study, our 360-degree evaluation system not only includes mutual evaluations between students and instructors, as well as assessments by nursing staff, but also introduces a new dimension: peer evaluation within the same group. Additionally, the evaluation criteria have been expanded to emphasize collaboration skills (e.g., collaboration during treatment and case compilation) and communication skills (including patient-clinician, student-instructor, and student-student interactions).

Traditional evaluation systems for undergraduate clinical internship have seldom incorporated assessments of clinical instructors. However, it is widely recognized that the behavior and teaching quality of instructors directly impact medical students (19, 20), while students' performance also reflects the instructional effectiveness of their supervisors (21). Therefore, feedback from students regarding their instructors is highly valuable. It enables hospital education administrators and departments to better understand the distribution of teaching competencies, allowing for targeted faculty development and overall improvement in teaching quality (22, 23). Based on this understanding, and inspired by the resident 360-degree evaluation system, we have—for the first time—incorporated student evaluations on clinical instructors into the assessment of undergraduate clinical training effectiveness.

In this study, the evaluations on the clinical instructors by students were generally high, with total scores of 90.63 and 91.37 out of 100 for the two instructors, respectively. This can be attributed to the fact that PKUSS, as a leading dental institution in China, maintains a high standard of teaching faculty. All clinical instructors undergo systematic training within a comprehensive faculty development pipeline, and are further supported by senior consultants with advanced professional titles, which enhances their overall teaching coordination capacity (12). The two instructors involved in this program were a senior attending physician, and a dentist with an associate senior professional title, both of whom have undergone systematic training in general dentistry and possess over 10 years of clinical teaching experience. They are highly skilled practitioners and have received awards in multiple national comprehensive case competitions. The absence of significant differences in the scores between the two instructors across all competency domains further confirms their comparable teaching proficiency.

The inclusion of two instructors was intended to avoid the potential rigidity of a single instructor's teaching approach. By exposing students to diverse teaching styles and perspectives, we aim to optimize training outcomes and enrich students' understanding of comprehensive patient care (17). Despite the high ratings from students, internal expert reviews still highlighted areas for improvement, such as increasing demonstration sessions and ensuring a balanced distribution of clinical procedures among students. This indicates that continuous enhancement of teaching organization and educational quality remains an ongoing and important challenge.

The results of this 360-degree evaluation reveal variations in students' performance across different competencies, indicating relative strengths and weaknesses in specific ability areas. These findings suggest that future medical training could benefit from a more targeted approach to enhance educational efficiency, which aligns with previous studies on 360-degree evaluations in medical education (24–27). When converted to a five-point scale, the scores show that the four items related to professional ethics received the highest ratings, reflecting the students' strong professional integrity, positive clinical attitude, proactive approach to work, and responsible patient care. This outcome is closely linked to our institution's sustained emphasis on humanistic education (13). In contrast, “*comprehensive workup and treatment planning*” under *Clinical Competence* received the lowest score of 3.97. Because this comprehensive clinical internship is often the first exposure for dental undergraduates to holistic patient management and integrated oral-health assessment, limited rotation time and relatively limited clinical experience may impede rapid skill acquisition in this complex area. Nevertheless, cultivating a generalist mindset and providing comprehensive, systematic patient care will remain essential throughout their professional development.

Comparative analysis of the common evaluation items rated by clinical instructors, nursing staff and peers revealed two areas with significant differences.

First, clinical instructors rated students' *collaboration skills* significantly higher than both nurse and peers (9.08 ± 1.11 vs. 7.82 ± 1.18 and 8.18 ± 1.02, *p* = 0.014 and 0.016, respectively). This discrepancy may be attributed to differences in role perspectives: clinical instructors tend to focus more on physician-specific responsibilities such as clinical examination, preoperative informed consent, diagnostic reasoning, and procedural skills. During treatment, their attention is often drawn to the performing student, potentially causing them to overlook the assisting student's level of engagement and collaboration. In contrast, the nurse and the performing student, who are more directly involved in auxiliary roles, may be better positioned to evaluate the assisting student's initiative and cooperation, thereby providing a more balanced assessment of collaboration skills.

Furthermore, the divergent scoring patterns among raters may stem from inconsistent conceptual definitions of collaboration ability across different evaluators. Meanwhile, such inter-rater differences also suggest that standardized rater training and unified evaluation criteria are insufficient at present. Targeted rater calibration training, clear operational definitions of collaborative behaviors, and refined scoring indicators can effectively narrow the scoring bias among different evaluators, enhance inter-rater reliability, and further improve the consistency and objectivity of MSF results in subsequent clinical teaching and evaluation practices.

Second, the nurse rated students' *communication skills* significantly lower than peers did (7.98 ± 0.89 vs. 9.01 ± 1.12, *p* = 0.010). Students admitted to PKUSS are among the top undergraduates in China—highly talented, confident, and enthusiastic, especially during their initial clinical experiences. They often exhibit strong motivation and warmth toward patients. However, due to limited experience in patient communication strategies, they may overestimate their own communicative effectiveness, which could partly explain the higher ratings from their peers (28). On the other hand, the nursing staff, who often takes responsibility for post-operative instructions and follow-up communications, are more involved in guiding and observing students' communication with patients. This places them in a better position to identify shortcomings in students' communication skills, which may account for the scoring gap between nurse and students—a finding consistent with that reported by Mahnaz et al. (26).

## 5 Limitations

Multi-source feedback (MSF) acts as a clinical and educational diagnostic instrument. Although pinpointing personal performance deficiencies is essential, the core objective of this assessment is to deliver targeted formative feedback for assessed students and guide their targeted professional growth. MSF outcomes provide students with detailed, multidimensional insights into their comprehensive clinical competence and professional behaviors, supporting their long-term continuous self-improvement and iterative ability optimization driven by assessment feedback (29). Furthermore, it helps identify gaps in comprehensive dental education activities. Accordingly, corresponding curriculum adjustment and teaching optimization strategies were formulated, constructing a full closed educational management loop covering assessment diagnosis, targeted rectification, teaching reform and effect re-verification, so as to steadily upgrade overall clinical comprehensive abilities of dental students. To achieve this, the 360-degree evaluation system must possess robust data analysis capabilities and effective methods for presenting results to enable stratified and targeted feedback (28–30).

Currently, the evaluation of clinical instructors is limited to numerical scores, without open-ended questions to gather more detailed student' suggestions. Moreover, the lack of significant differentiation in the ratings may indicate that the evaluation items are not sufficiently detailed, specific, or comprehensive. Due to the consistency of teaching arrangements, only two instructors were recruited in this study. Nevertheless, a larger teacher sample is required to draw solid conclusions regarding the reliability of instructor evaluation. This limitation also points out a worthwhile direction for our future teaching evaluation research. Accordingly, we frame the instructor evaluation component as a pilot or a feasibility study within the broader implementation of multi-source feedback (MSF).

So far, this study has only reached the stages of diagnosis and feedback. Future efforts should focus on tracking improvements, refining the evaluation scale to make it more scientific, concise, and targeted, as well as leveraging efficient information technology and designing smoother, more rational processes. These represent key directions for subsequent research.

It is important to note that clinical internships are not merely educational activities but also clinical practice. As such, patient satisfaction represents a critical yet overlooked metric for evaluation. This aspect was not addressed in our study and thus represents a notable limitation that should be considered in further research. To address it, patient feedback could be integrated through a structured, multi-faceted approach. Specifically, future research could incorporate patient-reported outcome measures (PROMs) tailored to dental education contexts, such as validated questionnaires assessing patient satisfaction with student clinicians' communication skills, empathy, and technical care. However, several practical barriers may hinder the integration of patient feedback. These include time constraints for patients who may be unwilling to complete additional assessments after dental visits, as well as the potential variability in patients' ability to objectively evaluate student performance, as their judgments may be influenced by personal experiences or limited understanding of dental professional standards.

The 360-degree evaluation model was implemented for a relatively short duration, spanning only 1 year, with only one cohort of 2020. The limited study period restricts the ability to draw definitive conclusions, and further evaluation is necessary to assess the long-term outcomes and impacts on dental education.

## 6 Conclusion

The 360-degree evaluation system enables a multi-perspective, continuous, and comprehensive assessment of both undergraduate dental students' competencies and the teaching abilities of instructors. Although dental students entering clinical internships demonstrate strong professionalism, there remains room for improvement in areas such as comprehensive diagnosis and treatment, teamwork, and communication skills. By analyzing feedback, targeted strengthening training can be provided more effectively. Moving forward, establishing a closed-loop management process of evaluation-improvement-verification will not only facilitates continuous improvement among dental students and strengthens their professional competence, but also promotes mutual growth between students and instructors, which in turn enhances the quality and outcomes of clinical training.

## Data Availability

The original contributions presented in the study are included in the article/supplementary material, further inquiries can be directed to the corresponding author.
